# Congenital Aural Stenosis: Clinical Features and Long-term Outcomes

**DOI:** 10.1038/srep27063

**Published:** 2016-06-03

**Authors:** Chen-long Li, Ying Chen, Yong-zheng Chen, Yao-yao Fu, Tian-yu Zhang

**Affiliations:** 1 Eye & ENT Hospital, Fudan University, Department of Otolaryngology-Head and Neck Surgery, Shanghai, 200032, China; 2 National Ministry of Public Health, Hearing Medicine Key Laboratory, Shanghai, 200032, China

## Abstract

The aim of the present study was to comprehensively evaluate the clinical features and long-term outcomes of congenital aural stenosis (CAS). This study presents a retrospective review of patients who underwent meatoplasty for CAS at a tertiary referral hospital from 2008 to 2015. A total of 246 meatoplasty procedures were performed on 232 patients in the present study. We performed multivariate regression analysis. Except in the age < 6 years group, no significant difference was observed among different age groups for cholesteatoma formation, p > 0.05. Except for the stenosis of the external auditory canal (EAC) (>4 mm) group, the other stenosis of EAC groups were not associated with cholesteatoma formation, p > 0.05. Postoperative air-bone gaps (ABG) less than 30 dB occurred in 77.3% (99/128) of the patients, and the Jahrsdoerfer score was associated with postoperative ABG, p < 0.001. The complication rate of CAS was 13.8% (20/144), and males showed a higher risk for postoperative complications (OR, 6.563; 95% CI, 1.268–33.966, p = 0.025). These results indicate that meatoplasty was an effective surgical intervention for CAS, showing a stable hearing outcome with prolonged follow-up. There was no significant difference between the cholesteatoma and no cholesteatoma groups for hearing outcomes, p > 0.05.

Congenital aural stenosis (CAS) carries a much greater risk of cholesteatoma compared with congenital aural atresia (CAA). Cole and Jahrsdoerfer defined CAS as an external auditory canal (EAC) with a diameter of 4 mm or less that frequently occurs in conjunction with grade 1 and grade 2 microtia. This diameter was selected because none of the patients with canal openings larger than 4 mm developed cholesteatoma^1^,^2^. The diagnosis of CAS is based on clinical examination (anamnesis, physical and audiometric evaluation) and imaging, particularly high-resolution computed tomography (HRCT) of the temporal bones^3^. The treatment of EAC cholesteatoma depends on the extent of the disease. Small lesions can typically be controlled with regular debridement, combined with the administration of topical antibiotics, while large and destructive lesions require surgery^4^.

The data on patients with CAS are limited, and the fundamental knowledge of CAS is still based on Cole and Jahrsdoerfer (1990). Two important parameters to consider when managing with CAS are patient age and stenosis size^1^. Patients with stenosis sizes of 2 mm or less are at high risk for developing cholesteatoma and should undergo surgery; however, the data in the present study challenged this viewpoint. To date, there are no large sample studies focusing on the clinical features and long-term outcomes of CAS, and no studies have described the important parameter of measuring the diameter of EAC^5^,^6^,^7^,^8^,^9^,^10^,^11^.

The aim of the present study was to comprehensively evaluate the clinical features and long-term outcomes of CAS, and challenge the previous viewpoint. Previous studies analysing the clinical features of CAS involved small sample sizes, and no studies focused on the stability of the hearing outcomes^5^,^6^,^7^. This study uses the largest sample of CAS, providing guidance in consultation and emphasizing important aspects for long-term follow-up.

## Results

A total of 246 meatoplasty procedures were performed on 232 patients in the present study. The mean age at surgery was 11.5 ± 7.2 years (range: 2–50 years), and 84.9% (209/246) of the patients were younger than 18 years when the procedures were performed. The mean time to follow-up was 18.3 ± 19.7 months (range: 1–78 months).

### Demographic data

For the purpose of this study, each of the 246 procedures was considered as an individual case, including 110 ears without cholesteatoma and 136 ears with cholesteatoma. In the cholesteatoma group, 53 ears were stages I and II, 55 ears were stage III and 28 ears were stage IV. There were 161 males with 97 cholesteatomas and 85 females with 39 cholesteatomas; 140 ears were affected on the right side with 90 cholesteatomas, and 106 ears were affected on the left side with 46 cholesteatomas; 57.3% (78/136) of the cholesteatoma patients also presented with infection, and 4.5% (5/110) of the patients without cholesteatoma presented with infection. To analyse the relationship between age and cholesteatoma formation, we divided the patients into four groups: age < 6, 6 ≤ age ＜ 12, 12 ≤ age ＜ 18 and age ≥ 18 years. Nearly half of the patients underwent surgery at 6 to 12 years old. A total of 32 meatoplasty procedures were performed in patients less than 6 years old. Except for 3 ears without cholesteatoma, the remaining 29 ears underwent surgery for cholesteatoma (Table 1). We conducted a multivariate logistic regression analysis for cholesteatoma formation. The results showed that males (OR, 2.401; 95% CI, 1.054–5.469, p = 0.037), the right side group (OR, 3.531; 95% CI, 1.618–7.706, p = 0.002); individuals aged <6 years (OR, 6.494; 95% CI, 1.263–33.396, p = 0.025), and the presence of infection (OR, 24.946; 95% CI, 8.432–73.803, p < 0.001) all showed a higher risk for cholesteatoma formation. Other age groups and degrees of microtia were not associated with cholesteatoma formation, p > 0.05.

### HRCT data

**Figure 1:**
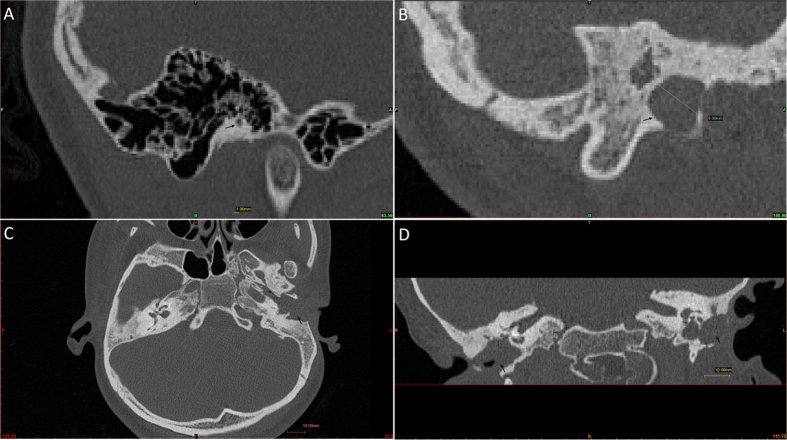
HRCT scans of CAS. (**A**) the right side stenotic EAC (black arrow) was 1 mm without cholesteatoma in an 8-year-old boy with CAS (sagittal view). (**B–D**) bilateral EAC cholesteatoma (black arrow) in a 17-year-old girl with CAS, the left side EAC was 9 mm (B, sagittal view; C, axial view; D, coronal view).

A total of 207 HRCT DICOM datasets were imported into MIMICS, including 94 ears without cholesteatoma and 113 ears with cholesteatoma. The mean stenosis of EAC at surgery was 3.1 ± 1.2 mm (range: 1–9 mm) (Fig. 1). A significant difference was observed between the cholesteatoma and no cholesteatoma groups for the stenosis of EAC using an unpaired t test, p < 0.01. A chi-square test also detected a significant difference among subgroups of stenotic EAC for cholesteatoma formation, χ^2^ = 12.4, p < 0.05, whereas the cholesteatoma group might exhibit a larger stenotic EAC reflecting the erosion of cholesteatoma. Among the 21 patients with cholesteatoma, individuals with stenosis of EAC (>4 mm) and a defective bony canal were observed. When we removed this group, there was no significant difference between the cholesteatoma and no cholesteatoma groups for the stenosis of EAC using an unpaired t test, p > 0.05, and the chi-square test detected no significant difference among the subgroups of stenotic EAC for cholesteatoma formation, χ^2^ = 2.5, p > 0.05. We conducted a multivariate logistic regression analysis for cholesteatoma formation. The results also show that the stenosis of EAC (>4 mm) group was associated with cholesteatoma formation (OR, 5.337; 95% CI, 1.444–19.735, p = 0.012), but the other stenosis of EAC groups were not associated with cholesteatoma formation, p > 0.05. These differences indicated that the stenosis of EAC group (>4 mm) with cholesteatoma might represent a special EAC state.

The mean Jahrsdoerfer score at surgery was 8.1 ± 1.6 (range: 4–10); the Jahrsdoerfer score for the no cholesteatoma group was 8.6 ± 1.2, and for the cholesteatoma group was 7.7 ± 1.7 (Table 2). A significant difference was detected between the cholesteatoma and no cholesteatoma groups in the Jahrsdoerfer scores using an unpaired t test, p < 0.01. For patients with more severe stages of EAC cholesteatoma, the Jahrsdoerfer score decreased, reflecting the erosion of cholesteatoma.

### Hearing outcomes

A total of 207 patients showed preoperative PTA, and 128 patients showed postoperative PTA. A total of 103 patients had complete data, including HRCT, preoperative and the latest postoperative PTA. Fifty-seven ears without cholesteatoma and 46 ears with cholesteatoma were analysed for hearing improvement (Table 3). There was no significant difference between the cholesteatoma and no cholesteatoma groups using an unpaired t test, including ΔABG, preoperative and postoperative ABG, p > 0.05.

We conducted a multivariate linear regression analysis for postoperative ABG. The results showed that the Jahrsdoerfer score was associated with postoperative ABG, p < 0.001; other factors, such as gender, age, laterality, presence of infection, cholesteatoma and stenosis of EAC, were not associated with postoperative ABG, p > 0.05. We also conducted a multivariate linear regression analysis for ΔABG. The results showed that the female group had better results for ΔABG, p = 0.032; other factors, such as age, laterality, presence of infection, cholesteatoma, stenosis of EAC and Jahrsdoerfer score, were not associated with ΔABG, p > 0.05.

**Figure 2:**
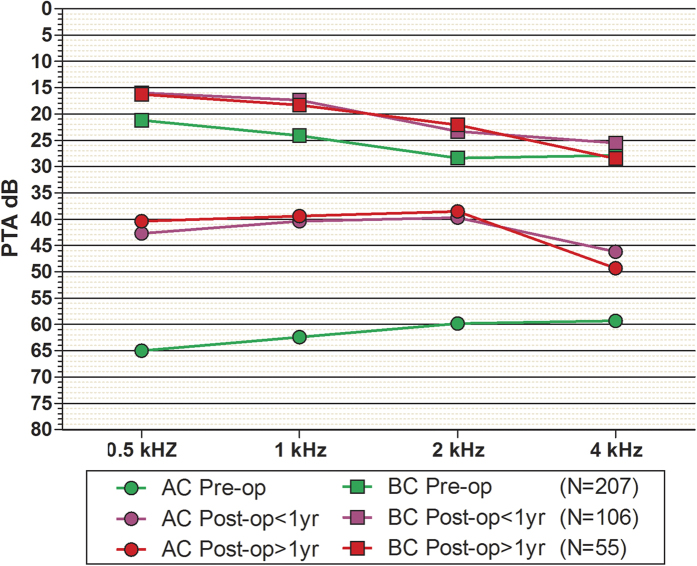
Long-term hearing results of PTA.

A latest postoperative ABG less than 30 dB was observed in 77.3% (99/128) of the CAS cases, and an ABG less than 10 dB was observed in 16.4% of the CAS cases (21/128) (Table 4). There was no significant difference between the cholesteatoma and no cholesteatoma groups in ABG < 30 dB (χ^2^ = 3.1, p > 0.05) or ABG < 10 dB (χ^2^ = 0.2, p > 0.05). To evaluate the stability of the hearing outcomes, a total of 106 patients underwent short-term (<1 yr) postoperative PTA, and 55 patients underwent long-term (>1 yr) postoperative PTA (Fig. 2). There was no significant difference between the short-term and long-term groups for postoperative PTA using a t test, p > 0.05. Nevertheless, we observed increased bone conduction (BC) hearing thresholds of 5 to 6 dB at 0.5, 1 and 2 kHz after surgery.

### Complication outcomes

We archived 144 patient records with effective postoperative follow-up, containing EAC and TM conditions and complications. The complication rate of CAS was 13.8% (20/144). None of the patients showed facial nerve palsy or total deafness. Six patients (4.1%) developed soft tissue stenosis, and 4 patients (2.7%) developed bony regrowth of the EAC. These two complications required further surgical procedures. Infection of the new EAC was observed in 1 (0.6%) patient, while the lateralization of TM was observed in 3 (2.0%) patients, the perforation of TM was observed in 5 (3.4%) patients and granulation tissue was detected in 1 (0.6%) patient. The complication rate in the cholesteatoma group was 20.5% (15/73), while the rate in the no cholesteatoma group was 7.0% (5/71) (Table 5). We conducted a multivariate logistic regression analysis for the complication rate. The results show that males had a higher risk for postoperative complication (OR, 6.563; 95% CI, 1.268–33.966, p = 0.025); other factors, such as age, laterality, presence of infection, cholesteatoma, stenosis of EAC and the Jahrsdoerfer score, were not associated with the complication rate, p > 0.05.

## Discussion

The problems associated with CAS include conductive hearing loss, cerumen impactions and EAC cholesteatoma^7^. The high risk of cholesteatoma formation suggests that more attention should be paid to patients with CAS. Cholesteatoma is a crucial factor in surgical indication, thus, patients were divided into cholesteatoma and no cholesteatoma groups in the present study.

Compared with other studies, this study included the largest sample of CAS patients with comprehensive analysis of the clinical features and long-term outcomes. There were significant differences between males and females, and between right and left sides for the formation of cholesteatoma. Males and right sides tended to show a higher risk of cholesteatoma. The male and right side predominances were consistent with the demographic data of microtia from different populations, and 80.0% (197/246) of the patients in the present study also presented with microtia^12^,^13^,^14^.

CAS patients without cholesteatoma typically underwent meatoplasty at ages greater than 6 years, when these patients were old enough to understand the rationale behind the meatoplasty procedure and assist in the postoperative care. Age was not an exclusion criterion in CAS patients with cholesteatoma, except patients aged <6 years, and there was no significant difference among different age groups for cholesteatoma formation. A previous study suggested that pediatric EAC cholesteatoma is less aggressive than adult, but age was not a crucial factor in surgical indication in the present study^15^. This result challenges the previous viewpoint that the appropriate time for surgery is late childhood or early adolescence, prior to the occurrence of irreversible damage^1^. There was some correlation between microtia and temporal bone malformation, evaluated using Marx grading systems^16^. Sixty-six percent of the individuals with deletions of the distal long arm of chromosome 18 had aural stenosis or atresia, and none of these subjects had microtia^17^. However, we also evaluated the relationship between the degree of microtia and EAC cholesteatoma, and there was no significant difference using the Marx grading system.

HRCT is indicated in patients with suspected congenital malformation of the external and middle ear, and preoperative planning is absolutely necessary^3^,^18^. The analysis was more accurate using MIMICS software^19^. The stenotic EAC was larger in the cholesteatoma group than in the no cholesteatoma group, reflecting the erosion of cholesteatoma. Among all 21 patients with cholesteatoma, the stenosis of EAC (>4 mm) group showed a defective bony canal. When we removed this group, there was no significant difference between the cholesteatoma and no cholesteatoma groups for the stenosis of EAC, and no significant difference was observed among the subgroups of stenotic EAC for cholesteatoma formation. We conducted a multivariate logistic regression analysis for cholesteatoma formation. The results also showed that the stenosis of EAC (>4 mm) group was associated with cholesteatoma formation (OR, 5.337; 95% CI, 1.444–19.735, p = 0.012), but the other stenosis of EAC groups were not associated with cholesteatoma formation, p > 0.05. These results challenged the previous viewpoint that patients with stenosis of 2 mm or less are at high risk of developing cholesteatoma and should undergo surgery^1^. Moreover, the differences in the results indicated that the stenosis of EAC group with cholesteatoma (>4 mm) might represent a special state of EAC, namely, the blockage of EAC. This state is a new concept based on HRCT, in which the EAC bony segment is expanded through cholesteatoma, and the stenotic EAC is larger than 4 mm. This result also challenged the previous viewpoint that CAS has been defined as an EAC with a diameter of 4 mm or less, as none of the patients with canal openings larger than 4 mm developed cholesteatoma^1^.

Except for some patients with stage IV EAC cholesteatoma, the middle ear was not eroded by cholesteatoma. Thus, there was no significant difference in the same surgical technique between the cholesteatoma and no cholesteatoma groups, including ΔABG, preoperative and postoperative ABG^20^. A previous study suggested that the Jahrsdoerfer score (modified) was less useful in terms of predicting long-term hearing prognosis after canal tympanoplasty for CAS in 25 patients^6^. Nevertheless, the mean Jahrsdoerfer score in the no cholesteatoma group was higher than that in the cholesteatoma group, and the Jahrsdoerfer score is a factor that affects postoperative ABG.

Modified meatoplasty with endoaural-conchal incision is an effective surgical intervention for CAS. In the present study, a stable hearing outcome was observed with prolonged follow-up. However, in CAA cases, the degradation of hearing outcomes was observed with prolonged follow-up. This difference might reflect the status of postoperative EAC, TM and ossicles^21^. Another interesting finding was increased BC hearing thresholds of 5 to 6 dB at 0.5, 1 and 2 kHz after surgery. Animal experiments demonstrated that the inertia of the ossicular chain contributed to partial BC hearing, and this inertia produced greater effects on high frequencies compared with low frequencies. Patients with CAS presented BC hearing loss, which could primarily reflect the absence of the inertia of the ossicular chain^22^.

None of the patients examined in the present study showed facial nerve palsy or total deafness. Postoperative complications, included soft tissue stenosis (4.1%), perforation of TM (3.4%), bony regrowth of EAC (2.7%), lateralization of TM (2.0%), granulation tissue (0.6%) and infection of the new EAC (0.6%). It has been suggested that normal epithelial migration from the tympanic membrane and EAC is an essential phenomenon to maintain the cleanliness of the outer ear. Predisposing factors, such as bony external ear irregularities and local inflammation, prevent or slow the normal migration of the squamous cells in the external ear canal, leading to a build-up of keratin debris, ultimately forming a cholesteatoma^18^,^23^,^24^. Males not only had a higher rate of cholesteatoma formation but also a higher rate of postoperative complications. The role of chronic inflammation during disease progression and recurrence might trigger cholesteatoma onset and are important in guiding clinical intervention^25^.

In conclusion, the results of the present study indicate that meatoplasty is an effective surgical intervention for CAS, and a stable hearing outcome was observed with prolonged follow-up. The Jahrsdoerfer score affected postoperative ABG, and age was not a crucial factor in surgical indication. Except for the stenosis of EAC (>4 mm) group, there was no significant difference among subgroups of stenotic EAC for cholesteatoma formation, and no significant difference between the cholesteatoma and no cholesteatoma groups for hearing outcomes was observed. The stenosis of EAC with cholesteatoma (>4 mm) might represent a special state of EAC, namely, the blockage of EAC. This blockage is a new concept based on HRCT, in which the EAC bony segment is expanded through cholesteatoma, and the stenotic EAC is larger than 4 mm.

## Methods

### Patient Selection

This study was a retrospective review of patients who underwent meatoplasty for CAS at a tertiary referral hospital, from April 2008 to August 2015. The inclusion criteria were patients with CAS who underwent meatoplasty. The exclusion criteria were acquired aural stenosis, otological surgery history, temporal bone fibrous dysplasia, or benign or malignant tumours in EAC. Reflecting the particularity of meatoplasty, most of the patients followed up regularly. Ultimately, a total of 246 meatoplasty procedures were performed on 232 patients who became the subjects in the present study. Fourteen patients were bilateral, and for the purpose of this study, each of the 246 procedures was considered an individual case. The study was approved through the institutional review board.

### Data Collection

A structured form was used to obtain patient data, including anamneses, pure-tone audiometry (PTA), HRCT of the temporal bones, operation notes and videos, pathology reports, postoperative follow-up records and detailed contact information. There is a complete medical record system at the hospital, and used a custom database software to process the data of CAS and CAA patients.

The demographic data for each patient, including age, gender, laterality, infection, stage of EAC cholesteatoma and degree of microtia, were collected. The CAS infections primarily manifested as otorrhea or postauricular subperiosteal abscess. The stage of EAC cholesteatoma was determined using the Naim classification, in which stages I and II could not easily be distinguished; therefore, we combined these stages together for analysis^26^. The degree of microtia was determined using Marx grading systems^27^.

HRCT images were reconstructed with 0.75 mm thick sections at 0.5 mm increments. Digital imaging and communication in medicine (DICOM) datasets were imported into MIMICS 15.0 software (Materialize, Belgium) for image processing^19^. This software enables the simultaneous viewing of HRCT datasets using a set of 2D images and a 3D rendered image for each dataset. The Jahrsdoerfer score, for preoperative evaluation and outcome expectations, was evaluated using MIMICS^28^. With respect to measuring the EAC diameter, an analytical method was used. Sagittal scans were used to measure the diameter of EAC on MIMICS; sagittal reconstruction is the most useful image for defining the involvement of anterior, posterior, and inferior walls by the EAC cholesteatoma^18^. Only bony segments were measured, reflecting actual CAS conditions, with or without cholesteatoma. The minor axis of EAC was measured in each slice, and the minimum value was considered as the stenosis size (Fig. 1).

PTA was performed in a soundproof booth. Frequencies of 0.5, 1, 2 and 4 kHz were analysed in the present study. We collected the preoperative and postoperative PTA and calculated the air-bone gaps (ABG), and the mean of the four frequencies was calculated as the ABG value^9^. The short-term (<1 yr) and long-term (>1 yr) hearing results were reviewed to evaluate the stability of the hearing outcomes^29^.

Postoperative complications were also collected during follow-up. We recorded the number and the type of different complications arising from meatoplasty, including stenosis, bony regrowth of EAC, infection, lateralization of tympanic membrane (TM), perforation of TM, granulation tissue of TM or EAC, total deafness and facial nerve palsy. Some patients showed more than one of these complications, and each of the complications was separately analysed in the present study.

### Surgical Technique and Follow-up

The same surgeon performed all procedures, and CAS patients without cholesteatoma typically underwent meatoplasty at ages greater than 6 years^1^. For CAS patients with cholesteatoma, age was not an exclusion criterion. We used a modified meatoplasty procedure with an endoaural-conchal incision, in which two local rotation flaps and a transposition split-thickness scalp flap were used to widen the stenotic EAC and reconstruct the TM. When necessary, i.e., Jahrsdoerfer score 6 or greater, tympanoplasty or ossicle mobilization was performed^20^,^30^. The long-term care of the ear is important because reconstructed EACs do not typically clear squamous debris^29^. Most of the patients required regular follow-up every 3 to 6 months. The postoperative follow-up records, including PTA, HRCT and complications, were archived using custom database software.

### Statistical Analysis

Descriptive and inferential statistical analyses were performed using parametric and nonparametric tests, as appropriate. For continuous variables, independent groups were compared using t tests. The relationships between categorical variables were assessed using χ^2^ analyses. Multivariate logistic/linear regression analysis were preformed to see whether CAS is independently associated with clinical features and long term outcomes. All analyses were performed using SPSS software (version 20.0; IBM, New York). For all comparisons and analyses, a p value of <0.05 was used as the cut-off point for statistical significance.

## Additional Information

**How to cite this article**: Li, C.-l. *et al.* Congenital Aural Stenosis: Clinical Features and Long-term Outcomes. *Sci. Rep.* **6**, 27063; doi: 10.1038/srep27063 (2016).

## Figures and Tables

**Table 1:** Demographic Data for the 246 Ears with CAS.

	Cholesteatoma				No cholesteatoma	Total
	Stage I & II	Stage III	Stage IV	Subtotal		
No.	53	55	28	136	110	246
Gender						
Male	38	42	17	97	64	161
Female	15	13	11	39	46	85
Laterality						
Left	18	17	11	46	60	106
Right	35	38	17	90	50	140
Age, mean ± SD, y	11.1 ± 7.3	10.8 ± 8.4	12.8 ± 8.0	11.3 ± 7.9	11.7 ± 6.2	11.5 ± 7.2
<6	12	13	4	29	3	32
6 ≤ Age ＜ 12	25	22	13	60	62	122
12 ≤ Age ＜ 18	5	15	5	25	30	55
≥18	11	5	6	22	15	37
Infection, n (%)	18 (33.9)	37 (67.2)	23 (82.1)	78 (57.3)	5 (4.5)	83 (33.7)
Microtia						
Marx 0	12	8	3	23	26	49
Marx I	23	22	12	57	48	105
Marx II	12	15	6	33	22	55
Marx III	6	10	7	23	14	37

**Table 2:** Characteristics of 207 Ears with CAS based on HRCT.

	Cholesteatoma				No cholesteatoma	Total (range)
	Stage I&II	Stage III	Stage IV	Subtotal		
No.	41	51	21	113	94	207
Stenosis of EAC, mean ± SD, mm	2.6 ± 1.0	3.5 ± 1.1	4.6 ± 1.5	3.4 ± 1.3	2.9 ± 1.0	3.1 ± 1.2 (1–9)
1 mm	7	2	0	9	6	15
2 mm	10	7	1	18	22	40
3 mm	14	15	5	34	39	73
4 mm	10	18	3	31	23	54
>4 mm	0	9	12	21	4	25
Jahrsdoerfer score, mean ± SD	8.2 ± 1.6	7.9 ± 1.5	6.1 ± 1.5	7.7 ± 1.7	8.6 ± 1.2	8.1 ± 1.6 (4–10)

**Table 3:** PTA at Cholesteatoma and No Cholesteatoma.

	Cholesteatoma (Jahrsdoerfer score)			Average (N = 46)	No cholesteatoma (Jahrsdoerfer score)			Average (N = 57
	≤8 (N = 17)	9 (N = 12)	10 (N = 17)		≤8 (N = 19)	9 (N = 19)	10 (N = 19)	
Preoperative ABG, mean ± SD, dB	34.1 ± 9.3	35.2 ± 10.4	30.9 ± 15.4	33.2 ± 12.0	37.5 ± 11.1	38.1 ± 11.0	33.4 ± 13.5	36.3 ± 11.9
Postoperative ABG, mean ± SD, dB	26.6 ± 12.2	19.5 ± 10.9	13.6 ± 7.4	20.0 ± 11.5	26.1 ± 8.0	18.3 ± 8.1	15.3 ± 9.3	19.9 ± 9.5
ΔABG(dB), mean ± SD, dB	7.5 ± 12.7	15.6 ± 15.1	17.2 ± 17.0	13.2 ± 15.3	11.3 ± 11.6	19.8 ± 13.0	18.1 ± 15.0	16.4 ± 13.5

**Table 4:** Hearing Sensitivity Recovery.

	ABG < 30 dB, n (%)	ABG < 10 dB, n (%)
Cholesteatoma (N = 61)	43 (70.4)	11 (18.0)
Jahrsdoerfer score ≤ 8 (N = 23)	13 (56.5)	1 (4.3)
Jahrsdoerfer score = 9 (N = 13)	10 (76.9)	3 (23.0)
Jahrsdoerfer score = 10 (N = 17)	16 (94.1)	7 (41.1)
No Cholesteatoma (N = 67)	56 (83.5)	10 (14.9)
Jahrsdoerfer score ≤ 8 (N = 20)	13 (65.0)	0
Jahrsdoerfer score = 9 (N = 19)	17 (89.4)	3 (15.7)
Jahrsdoerfer score = 10 (N = 19)	18 (94.7)	6 (31.5)
Total (N = 128)	99 (77.3)	21 (16.4)

**Table 5:** Complications Arising From Meatoplasty.

	Cholesteatoma (N = 73), n (%)	No Cholesteatoma (N = 71), n (%)	Total (N = 144), n (%)
Stenosis	4 (5.4)	2 (2.8)	6 (4.1)
Bony regrowth	2 (2.7)	2 (2.8)	4 (2.7)
Infection	1 (1.3)	0	1 (0.6)
Lateralization of TM	3 (4.1)	0	3 (2.0)
Perforation of TM	4 (5.4)	1 (1.4)	5 (3.4)
Granulation tissue	1 (1.3)	0	1 (0.6)
Total deafness	0	0	0
Facial nerve palsy	0	0	0
Total	15 (20.5)	5 (7.0)	20 (13.8)
